# The role of hepatitis B infection in anti-tuberculosis drug-induced liver injury: a meta-analysis of cohort studies

**DOI:** 10.1017/S0950268820002861

**Published:** 2020-11-23

**Authors:** Jing Zheng, Mei-Hong Guo, He-Wei Peng, Xiao-Ling Cai, Yun-Li Wu, Xian-E Peng

**Affiliations:** 1 Department of Epidemiology and Health Statistics, Fujian Provincial Key Laboratory of Environment Factors and Cancer, School of Public Health, Fujian Medical University, Fujian, China; 2 Key Laboratory of Ministry of Education for Gastrointestinal Cancer, Fujian Medical University, Fujian, China

**Keywords:** Anti-tuberculosis, hepatitis B infection, liver injury

## Abstract

Drug-induced liver injury (DILI) is a common adverse drug reaction leading to the interruption of tuberculosis (TB) therapy. We aimed to identify whether the hepatitis B virus (HBV) infection would increase the risk of DILI during first-line TB treatment. A meta-analysis of cohort studies searched in PubMed, Web of Science and China National Knowledge Infrastructure was conducted. Effect sizes were reported as risk ratios (RRs) and 95% confidence intervals (CIs) and calculated by R software. Sixteen studies with 3960 TB patients were eligible for analysis. The risk of DILI appeared to be higher in TB patients co-infected with HBV (RR 2.66; 95% CI 2.13–3.32) than those without HBV infection. Moreover, patients with positive hepatitis B e antigen (HBeAg) were more likely to develop DILI (RR 3.42; 95% CI 1.95–5.98) compared to those with negative HBeAg (RR 2.30; 95% CI 1.66–3.18). Co-infection with HBV was not associated with a higher rate of anti-TB DILI in latent TB patients (RR 4.48; 95% CI 0.80–24.99). The effect of HBV infection on aggravating anti-TB DILI was independent of study participants, whether they were newly diagnosed with TB or not. Besides, TB and HBV co-infection patients had a longer duration of recovery from DILI compared to non-co-infected patients (SMD 2.26; 95% CI 1.87–2.66). To conclude, the results demonstrate that HBV infection would increase the risk of DILI during TB therapy, especially in patients with positive HBeAg, and close liver function monitoring is needed for TB and HBV co-infection patients.

## Introduction

Tuberculosis (TB) is a chronic communicable disease caused by *Mycobacterium tuberculosis*. The WHO estimates that in 2018, 10 million people were affected by TB. Especially, the number of TB cases is larger in South-East Asia (44%), Africa (24%) and the Western Pacific (18%) [1]. Among single infectious pathogen illnesses, a high proportion of deaths results from TB, which remains a threatening public health issue worldwide [2, 3]. As a recommended anti-TB strategy, short-course first-line medication regimens have been proved to be effective [1]. Isoniazid is of great importance to kill bacilli during the early anti-TB period. With the addition of pyrazinamide, the sterilizing effect gets better [4, 5]. Rifampicin plays a pivotal role in declining non-replicating bacteria and reduces the TB relapse rate [6]. However, drug-induced liver injury (DILI) associated with the above medications is a common adverse drug reaction. Hepatotoxicity is likely to develop further severely when used in combination, especially on the addition of pyrazinamide [7–9]. Thus, anti-TB DILI would result in the interruption of chemotherapy regimens and even cause death.

Without novel agents identified to replace first-line drugs, risk factors should be taken into consideration when preventing the occurrence of DILI. The regions with high endemicity for TB largely overlap that with a high hepatitis B virus (HBV) prevalence, especially in Asia and Africa [10]. Many epidemiological studies were conducted to investigate whether HBV infection can increase the incidence and severity of anti-TB DILI, but the results remain inconsistent [11, 12]. Recently, a meta-analysis reported that articles have indicated that HBV infection may increase the risk of DILI in the therapy for active TB [13]. However, articles included in the study were only published in English language, and multiple subgroup analyses were not carried out to explore the clinical variability. Given the high burden of TB and HBV infection, the situation of China attracts much attention. In China, most researches conducted by the front-line physician on the subject are published in Chinese. Therefore, the previous meta-analysis results may be subject to potential publication bias. We will perform a meta-analysis to estimate whether HBV infection would increase the risk of DILI during first-line TB treatment based on cohort studies. Furthermore, subgroup analyses were conducted to identify the sources of heterogeneity.

## Methods

The present meta-analysis was conducted in accordance with the PRISMA 2015 guideline [14].

### Literature search strategy

To search for the cohort studies of HBV infection in relation to DILI risk during TB treatment, a literature search was conducted on 20 June 2019 and updated on 23 February 2020 in PubMed, Web of Science and China National Knowledge Infrastructure. The following medical subject heading terms were used: ‘anti-tuberculosis’ OR ‘tuberculosis treatment’ OR ‘TB treatment’ AND ‘chronic hepatitis B’ OR ‘hepatitis B virus’ OR ‘HBV’. Additional articles were retrieved by a manual review of reference lists from the relevant original and review articles.

### Inclusion and exclusion criteria

Articles included in the current meta-analysis meet the following criteria: (1) the study design was the cohort study with concurrent comparison groups; (2) first-line medications had been used for active TB treatment, and isoniazid had been used for latent TB treatment; (3) participants had normal baseline liver function before starting TB treatment; (4) HBV carriers were identified as positive hepatitis B surface antigen (HBsAg), and DILI was defined as an increase in serum alanine aminotransferase (ALT) ⩾2 times the upper limit of normal (ULN; normal level ⩽40 IU/l).

Inversely, the exclusion criteria were: (1) review article, abstract, comments, letters, article with no quantitative information or details; (2) without the definition of DILI; (3) antiretroviral or hepatoprotective therapy combined with TB treatment; (4) more than 15% participants lost to follow-up.

Two investigators (JZ and MHG) independently reviewed each abstract and selected articles for the full review. The same inclusion and exclusion criteria were applied.

### Data extraction and quality assessment

Data were collected independently by two investigators (JZ and MHG), and the discrepancies were resolved by consulting with a third independent investigator (HWP). The extracted data contained both qualitative and quantitative information. In particular, qualitative information included the last name of the first author, year of publication, type of study design, the region where the study was conducted, anti-TB regimens and primary definition of DILI. Quantitative data included the number of patients with liver cirrhosis and DILI in the HBV co-infection group and non-co-infection group.

Quality assessment of each included study was based on the Newcastle-Ottawa Scale, a nine-star scoring system [15]. For cohort studies, representation of the exposed group, the selection method of non-exposed, ascertainment of exposure, lack of outcome at baseline, comparability of groups, assessment method, enough long follow-up period and adequacy of follow-up visits were assessed. We define ⩾7 points as good quality, 6 points as fair quality and ⩽5 points as poor quality [16]. The two investigators independently scored the methods sections and settled the differences by consensus.

### Statistical analysis

The number of eligible patients who were co-infected or not co-infected with HBV was abstracted or derived using data reported in the studies. The effect size was estimated by risk ratios (RRs) with its 95% confidence intervals (CIs). Studies were weighted by the Mantel–Haenszel method. We examined possible heterogeneity in results across studies using the *I*^2^ statistic and *χ*^2^ test. *I*^2^ ⩾ 50% or *P* < 0.10 according to the *Q* statistic, which was considered to be of substantial heterogeneity, and a random-effect model was used to calculate the pooled effect size. Otherwise, a fixed-effect model was used. When there was significant heterogeneity, subgroup analyses, and meta-regression analyses based on paper language, DILI definition, study design, sample size and study participants, and TB category were conducted. Subgroup analyses could also be used to investigate clinical variability.

Furthermore, a sensitivity analysis was performed by removing each study individually to evaluate the stability and reliability of the results of the primary meta-analysis. As there were at least 10 studies included in the meta-analysis, publication bias was detected by the funnel plot and Egger's linear regression test. All statistical analyses were conducted using R software (Version 3.5.3), and *P* < 0.05 was considered statistically significant, except where noted.

## Results

### Literature search

**Fig. 1.**
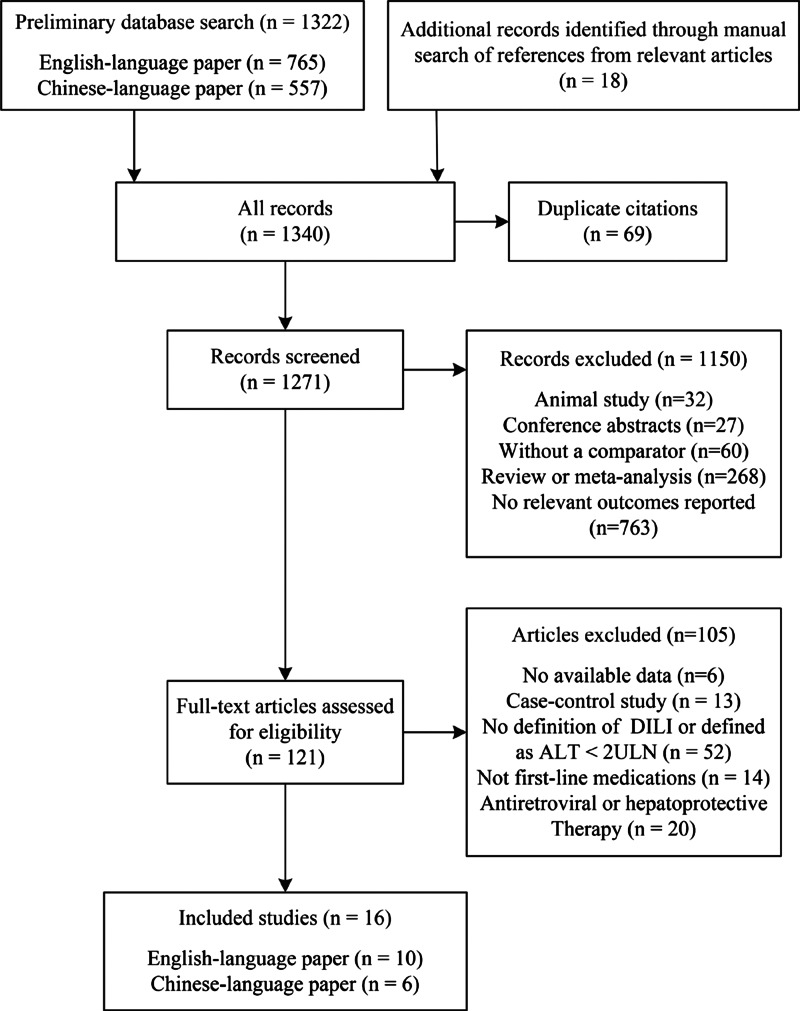
Flow diagram of the study selection process.

Figure 1 shows the study selection process. We identified 1322 publications through the search of different databases. Eighteen additional articles were identified through a manual search of reference lists from the relevant original and review articles. Finally, we screened 121 full-text articles and selected 16 articles for inclusion in the meta-analysis, including 10 English-language papers and six Chinese-language papers [11, 12, 17–30].

### Study characteristics

The baseline characteristics of the included studies are listed in Table 1. Of the 3960 patients who underwent TB treatment, 936 individuals with HBV infection and 3024 controls without HBV infection were enrolled in this analysis. Anti-TB regimens were based on isoniazid, rifampicin, pyrazinamide and ethambutol, except for two studies focused on isoniazid [17, 18]. A small proportion of participants were lost to follow-up in four studies [17, 18, 24, 28]. Eight of the 16 studies were prospective cohort studies, and the other eight studies were retrospective cohort studies. Eight of the studies focused on patients newly diagnosed with TB, and the other eight studies were not. Six studies adopted the stricter definition of DILI as serum ALT more than five times the ULN, while the other 10 studies used a looser definition of DILI (ALT ⩾ 2 or 3 ULN).

**Table 1.** Characteristics and quality scores of the included studies in the order of publication year

First author, year	Region	Study design	No HBV infection		HBV infection		Anti-tuberculosis regiments	Main definition of liver injury	Quality score
			No. of liver cirrhosis	No. of events/total	No. of liver cirrhosis	No. of events/total			
Patel, 2002	Iowa, America	Prospective	0	0/94	0	3/50	Isoniazid	ALT > 5 ULN	6
Fernandez-Villar, 2003	Vigo, Spain	Prospective	0	20/406	0	0/8	Isoniazid	ALT > 5 ULN	6
Pan, 2005	Shaanxi, China	Prospective	0	34/91	0	19/24	First-line drugs	ALT > 33.4 ULN	7
Anand, 2006	Pune, India	Prospective	0	13/128	0	9/24	First-line drugs	ALT > 5 ULN	7
Chien, 2010	Taiwan, China	Retrospective	0	22/270	0	3/25	First-line drugs	ALT ⩾ 5 ULN	7
Wang, 2011	Taiwan, China	Prospective	16	42/294	1	16/42	First-line drugs	ALT > 5 ULN	7
Chang, 2013	Henan, China	Retrospective	0	22/130	0	104/136	First-line drugs	ALT ⩾ 2 ULN	9
Xu, 2013	Guangdong, China	Prospective	0	9/87	0	26/82	First-line drugs	ALT > 2 ULN	8
Zhang, 2013	Guangxi, China	Retrospective	0	3/86	0	22/86	First-line drugs	ALT > 2 ULN	7
Cao, 2014	Shandong, China	Retrospective	0	23/95	0	82/90	First-line drugs	ALT > 2 ULN	9
Tang, 2015	Guizhou, China	Prospective	0	12/85	0	19/62	First-line drugs	ALT ⩾ 2 ULN	9
Isa, 2016	Jos, Nigeria	Retrospective	0	13/75	0	1/9	First-line drugs	ALT > 3 ULN	7
Kim, 2016	Jinju, Korea	Retrospective	3	25/251	21	11/83	First-line drugs	ALT > 3 ULN	9
Sun, 2016	Shanghai, China	Prospective	0	96/842	0	25/96	First-line drugs	ALT > 3 ULN	6
Tang, 2016	Yunnan, China	Retrospective	0	10/64	0	32/61	First-line drugs	ALT > 2 ULN	9
Chen, 2018	Guangdong, China	Retrospective	2	10/26	32	39/58	First-line drugs	ALT ⩾ 3 ULN	7

HBsAg, hepatitis B surface antigen; ULN, the upper limit of normal.

### Quality assessment

The quality assessment of the eligible studies is summarised in Table 1. Five articles scored nine points; one article scored eight points; seven articles scored seven points; three articles scored six points. Common potential sources of bias included lacking information on confounding factors controlling and characteristics comparability of participants who lost to follow-up between the two study groups.

### Meta-analysis

**Fig. 2.**
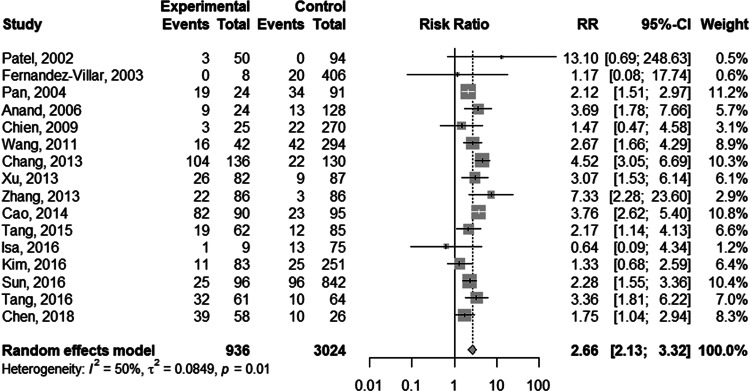
Forest plot of the association between HBV infection and the risk of anti-TB DILI.

**Fig. 3.**
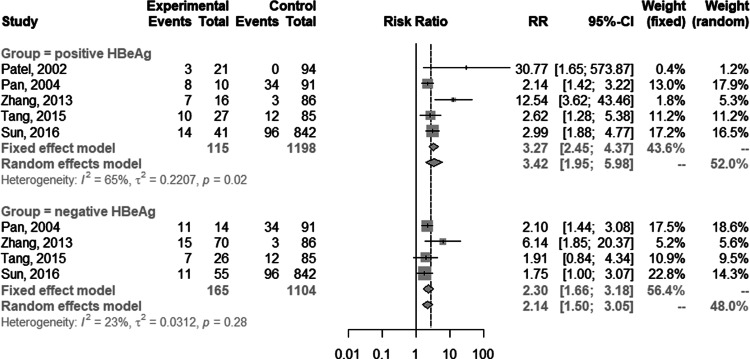
Forest plot of the association between HBV infection with different HBeAg status and the risk of anti-TB DILI.

The random-effect model was selected for the significant heterogeneity (*I*^2^ = 50%, *P* = 0.01) of 16 studies. Overall, TB patients with HBV infection were more likely to develop DILI (RR 2.66; 95% CI 2.13–3.32; Fig. 2) than those without HBV infection. Further analyses were performed on HBV infection patients with positive and negative hepatitis B e antigen (HBeAg) individually. Five studies provided data on positive HBeAg patients. The random-effect model (*I*^2^ = 65%; *P* = 0.02) showed the pooled RR of positive HBeAg developed to DILI was 3.42 (95% CI 1.95–5.98; Fig. 3). The same five studies provided the data of negative HBeAg patients; however, an RR estimate was not calculable in one study as no patient developed DILI in either group. Thus, four studies were pooled to give an RR of 2.30 (95% CI 1.66–3.18; Fig. 3) by the random-effect model (*I*^2^ = 23%; *P* = 0.28).

### Subgroup analysis and meta-regression analysis

Table 2 displays the main results of subgroup analyses. The pooled RR was 2.08 (95% CI 1.72–2.52) and 3.78 (95% CI 3.05–4.69) in the studies with English-language and Chinese-language paper separately. HBV infection showed a higher risk of anti-TB DILI in the group with a loose definition of DILI (RR 3.78; 95% CI 3.05–4.69) than in the other two groups with a strict definition of DILI (RR 1.79; 95% CI 1.36–2.37; RR 2.50; 95% CI 1.92–3.24). No significant heterogeneity was found in prospective cohort studies (*I*^2^ = 0%, *P* = 0.73). The fixed-effect model showed the pooled RR was 2.52 (95% CI 2.05–3.09), while the pooled RR was 2.68 (95% CI 1.78–4.04) by using the random-effect model in retrospective cohort studies. The similar effect size was detected in the patients whether they were newly diagnosed with TB (RR 2.76; 95% CI 2.16–3.52) or not (RR 2.65; 95% CI 1.89–3.72), as well as in the studies with different sample sizes (RR 2.68; 95% CI 2.14–3.37; RR 2.67; 95% CI 1.89–3.75). Besides, the fixed-effect model revealed that co-infection with HBV was not associated with a higher rate of anti-TB DILI in latent TB patients (RR 4.48; 95% CI 0.80–24.99).

**Table 2.** Main results of subgroup analysis and meta-regression analysis

Group	Included studies	Heterogeneity			Analysis model	Effect size	
		*I*^2^ statistic: (%)	*Q* statistic	*P-*value		RR	95% CI
Paper language							
English	10	3	9.31	0.41	Fixed-effect model	2.08	1.72–2.52
Chinese	6	7	5.37	0.37	Fixed-effect model	3.78	3.05 −4.69
DILI definition							
2 ULN ⩽ ALT < 3 ULN	6	7	5.37	0.37	Fixed-effect model	3.78	3.05–4.69
3 ULN ⩽ ALT < 5 ULN	4	12	3.40	0.33	Fixed-effect model	1.79	1.36–2.37
ALT ⩾ 5 ULN	6	0	4.44	0.49	Fixed-effect model	2.50	1.92–3.24
Study design							
Prospective	8	0	4.39	0.73	Fixed-effect model	2.52	2.05–3.09
Retrospective	8	68	22.13	<0.01	Random-effect model	2.68	1.78–4.04
Sample size							
<250	9	36	12.41	0.13	Fixed-effect model	2.68	2.14–3.37
⩾250	7	62	15.69	0.02	Random-effect model	2.67	1.89–3.75
Study participants							
Newly diagnosed with TB	8	11	7.89	0.34	Fixed-effect model	2.76	2.16–3.52
Not newly diagnosed with TB	8	68	21.89	<0.01	Random-effect model	2.65	1.89–3.72
TB category							
Latent TB	2	31	1.45	0.23	Fixed-effect model	4.48	0.80–24,99
Active TB	14	54	28.31	<0.01	Random-effect model	2.65	2.11–3.32

RR, risk ratio; CI, confidence interval; ULN, the upper limit of normal; TB, tuberculosis.

Given the substantial heterogeneity among the included studies, subgroup analysis and meta-regression analysis were used to explore the explanations of heterogeneity. As studies were stratified by paper language and definition of DILI, decreased *I*^2^ values were shown in all subgroups. Combined with meta-regression analysis results, the heterogeneity of the included studies may mainly relate to paper language and DILI definition (*P* < 0.001).

### Duration of recovery from DILI

**Fig. 4.**
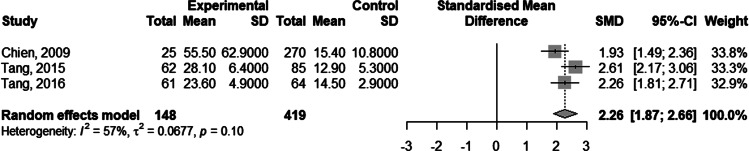
Forest plot of the duration of recovery from anti-TB DILI in HBV carriers compared to non-carriers.

Four studies [12, 21, 27, 29] showed a longer duration of recovery from DILI in HBV co-infected patients compared to non-co-infected patients (55.5 ± 62.9 *vs.* 15.4 ± 10.8 days [21]; 28.1 ± 6.4 *vs.* 12.9 ± 5.3 days [27]; 23.6 ± 4.9 *vs.* 14.5 ± 2.9 days [29]). One study presented the Kaplan–Meier curve for recovery from DILI in the two groups without reporting the recovery time. A meta-analysis on three related studies was performed to explore the pooled effect. The result showed the standardised mean difference in DILI recovery duration of patients with and without HBV co-infection was 2.26 (95% CI 1.87–2.66; Fig. 4).

### Sensitivity analysis and publication bias test

**Fig. 5.**
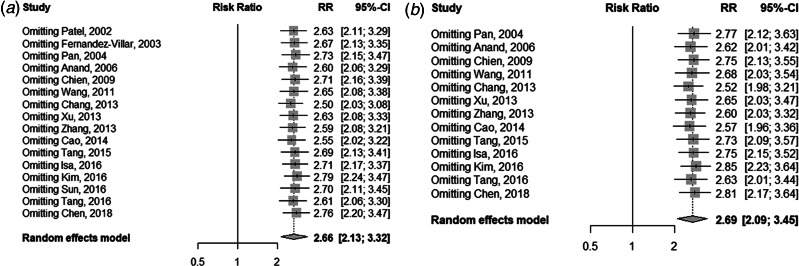
Sensitivity analysis of the association between HBV infection and the risk of anti-TB DILI. (a) For all including studies; (b) excluding the fair-quality studies.

**Fig. 6.**
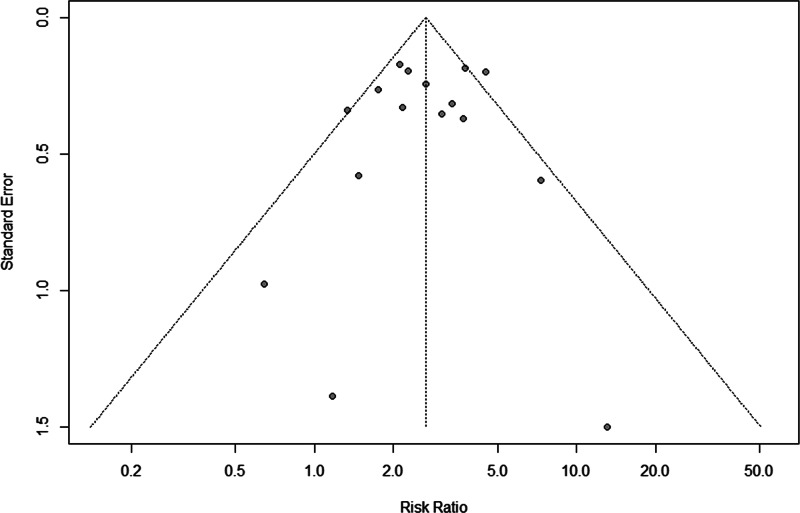
Funnel plot for the assessment of publication bias.

Figure 5 displays the sensitivity analysis results. Pooled RRs were calculated after the exclusion of one study at a time. These analyses did not reveal any notable changes in the estimates, with RRs for HBV infection and anti-TB DILI between 2.50 and 2.79. Besides, when the fair-quality studies were removed, there was no significant change. As Figure 6 showed, the funnel plot did not suggest any publication bias with the *P*-value for the Egger's test being 0.707.

## Discussion

HBV infection is a recognised risk factor for liver injury [31]. The relationship between HBV infection and the risk of anti-TB DILI remains to be explored. On analysing the pooled effect size from 16 studies, the risk of DILI appeared to be higher in TB patients co-infected with HBV than those without HBV infection. Meanwhile, four studies reported that the duration of recovery from DILI was longer in HBV co-infected patients, indicating that HBV carriers might develop DILI more severely. Furthermore, HBV infection patients with positive HBeAg were more likely to develop DILI compared to those with negative HBeAg. HBeAg is closely related to HBV replication activity so that the status of HBeAg may aggravate the interference effect of viral infection during TB therapy. The association between viral load and the risk of anti-TB DILI was not performed for the insufficient data in the included studies. Zhu *et al*. suggested that high levels of HBV-DNA might be a risk factor for anti-TB DILI, although the odds ratio value was 2.066, which did not achieve statistical significance [32].

In the subgroup analyses, HBV infection showed a higher risk of anti-TB DILI in the group with a loose definition of DILI than in the other two groups with a strict definition of DILI. The diagnosis of DILI remains a challenge, and no recognised tests were available for physicians to establish the diagnosis [33]. Hence, in our study, ALT elevation in serum was used to determine whether patients will develop serum bilirubin elevation, indicating systemic DILI. When DILI was defined as asymptomatic ALT ⩾5 ULN, the pooled RR was 2.50 with six studies. Additionally, the risk of DILI was similar in groups with different sample sizes, and the pooled effect was independent of study participants whether they were newly diagnosed with TB. There was no heterogeneity detected in the eight prospective cohort studies, which may result from closer liver function monitoring. As for the retrospective cohort studies included in our meta-analysis, all medical records were available and reviewed in detail. Furthermore, co-infection with HBV was not associated with a higher rate of anti-TB DILI in latent TB patients.

Significant heterogeneity of the included studies can be noticed, it may be explained by paper language and definition of DILI. In view of studies with English-language paper, all used a stricter DILI diagnostic criterion. Therefore, the heterogeneity may mainly relate to the DILI definition. Besides, the sensitivity analysis suggested that the pooled effect with 16 studies was robust, and no significant publication bias was detected by the funnel plot (*P* > 0.05).

Thus far, the shared pathogenetic mechanisms of HBV infection and anti-TB regimens inducing liver injury remain unclear. Cao *et al*. insisted that anti-TB drugs could generate various metabolites, which changed with the liver state of the body [34]. T-cell-dependent immune responses would be triggered by drugs or their metabolites, interacting with immune receptors [35]. The presence of viral infection-induced hepatocyte stress might contribute to the immune responses by raising the levels of cytokines and cell-surface markers and the expression of costimulatory molecules, which can increase the frequency of drug hypersensitivity reactions [36]. When HBV carriers are combined with positive HBeAg or high levels of HBV-DNA, proinflammatory cytokines and chemokines could be provoked to overexpress by actively replicating virus, which might aggravate the risk of DILI [37]. Besides, HBV infection would enhance lipid peroxidation and induce reactive oxygen species accumulation due to anti-TB drugs. Further, oxidative stress destroys the normal infrastructure and activities of cells and promotes apoptosis [38, 39].

Compared to the previous study [13], the present meta-analysis showed a similar finding that HBV co-infection might increase the risk of DILI in the first-line medication therapy for TB. Differently, our analysis only included cohort studies for the higher causal validation power and lower information bias. We used a stricter definition of DILI and provided a richer set of findings, which may facilitate providing references for clinical decision-making. Moreover, there is recent evidence that prophylactic antiviral therapy for HBV could reduce the incidence of DILI in patients with TB-HBV co-infection [40, 41]. A sequential rather than an incremental approach should be administered to reduce the probability of hepatotoxicity during TB therapy [42].

Several factors limited the present study. First, the studies included in our analysis were all observational cohort designs without selecting study subjects randomly so that it is difficult to avoid potential selection bias and confounding factors. Second, as a majority of studies lacking data for HBeAg status in HBV carriers to develop DILI, the finding needs to be interpreted with caution. Third, although we used a stricter definition of DILI than the previous meta-analysis, it is difficult to avoid including patients with hepatic adaptation to anti-TB treatment since different studies used different definition criteria. We performed a subgroup analysis with studies using a strict *vs.* loose definition of DILI which may help to mitigate this defect to some extent.

In conclusion, our meta-analysis with a large sample size showed that HBV infection would increase the risk of DILI during TB therapy. Based on our findings, TB and HBV co-infection patients with positive HBeAg may have a higher risk of anti-TB DILI than those with negative HBeAg, and close liver function monitoring is needed for HBV co-infection patients whether they were newly diagnosed with TB or not.

## Data

Data sharing is not applicable to this article as no new data were created or analysed in this study.
